# Supplementary material for: Identification of Freezing-Responsive microRNAs and Their Targets in Chinese Jujube by Small RNA and Degradome Sequencing
Source: Int J Mol Sci. 2026 Sep 4;27(17):7912. doi: 10.3390/ijms27177912 (PMC13566890; doi:10.3390/ijms27177912)
Supplement: Supplementary file 1 [file ijms-27-07912-s001.zip › Supplementary Material S2/novel_miR_15.pdf]

Provisional ID	:	novel_miR_15
Score total	:	0.4
Score for star read(s)	:	-1.3
Score for read counts	:	0
Score for mfe	:	0.1
Score for randfold	:	1.6
Score for cons. seed	:	
Total read count	:	656
Mature read count	:	656
Loop read count	:	0
Star read count	:	0

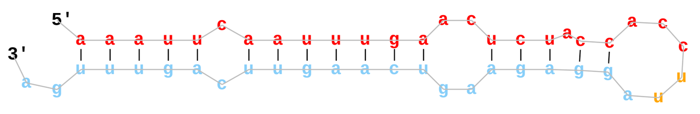

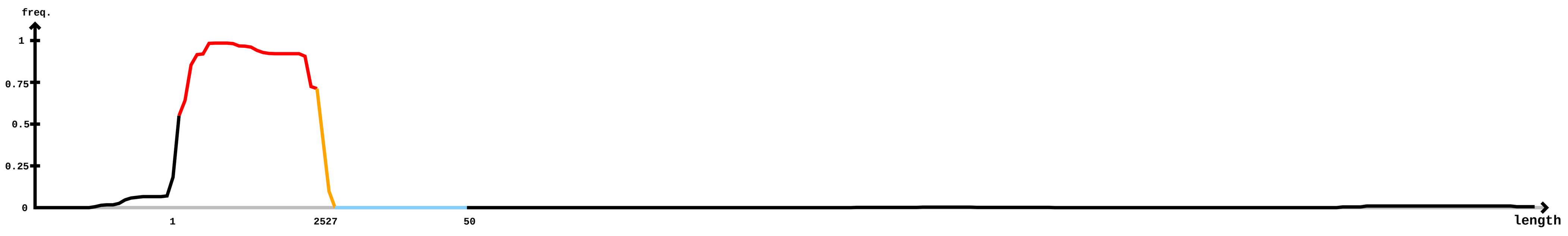

Mature Star

```
5'- agaaucuaucaugauagaggacaaauucaauuugaacucuaccaccuuaggagaagucaaguucaguuugaacucuacuuuagaagaauucaauuucaaguugaacucuaccaaaggcgaauucaauuucaauuugaauuccaccaccucuucuccuuuauauauaugcauuuaggcuuauuguaaaauuugaucccauuuuguaaaaagagagagcuuuaauucucuuccucucaauuauauugucuug -3'   exp
    (((.((((....(.(((((..((((((.(((((((..(((.((......)))))..))))))).))))))..))))))..)))).((((((((.((.((((((((.((..((...)).)).)))))))).)).))))))))..............((((((.(((.((......)).)))))))))......................(((((((......)))))))......((((...))))))..   reads   mm   sample
    ........ucauUauagaggacaaauuc..............................................................................................................................................................................................................................   2   1   LT-1
    ........ucaugauagaggacaaauucaauu..........................................................................................................................................................................................................................   2   0   LT-1
    .........caugauagaggacaaauucaau...........................................................................................................................................................................................................................   2   0   LT-2
    .........caugauagaggacaaauucaauu..........................................................................................................................................................................................................................   3   0   LCK-3
    .........Uaugauagaggacaaauucaauu..........................................................................................................................................................................................................................   1   1   LCK-3
    ..........augauagaggacaaauucaauug.........................................................................................................................................................................................................................   1   0   JCK-3
    ..........augauagaggacaaauucaauug.........................................................................................................................................................................................................................   1   0   JCK-2
    ............gauagaggacaaauucaauuuga.......................................................................................................................................................................................................................   1   0   JCK-2
    ............gauagaggacaaauucaauuuga.......................................................................................................................................................................................................................   1   0   LT-2
    ............gauagaggacaaauucaauuugaa......................................................................................................................................................................................................................   1   0   JCK-1
    ............gauagaggacaaauucaauuugaa......................................................................................................................................................................................................................   3   0   JCK-2
    .............auagaggacaaauucaauu..........................................................................................................................................................................................................................   2   0   LT-1
    .............auagaggacaaauucaUuu..........................................................................................................................................................................................................................   2   1   LCK-3
    .............auagaggacaaauucaauuu.........................................................................................................................................................................................................................   1   0   LT-3
    .............auagaggacaaauucaauuuga.......................................................................................................................................................................................................................   1   0   JCK-1
    .............auagaggacaaauucaauuuga.......................................................................................................................................................................................................................   3   0   JCK-3
    .............auagaggacaaauucaauuuga.......................................................................................................................................................................................................................   5   0   JCK-2
    .............auagaggacaaauucaauuugaU......................................................................................................................................................................................................................   1   1   LT-2
    ..............uagaggacaaauucaauuug........................................................................................................................................................................................................................   2   0   JCK-2
    ..............uagaggacaaauucaauuuga.......................................................................................................................................................................................................................   2   0   LT-3
    ..............uagaggacaaauucaauuugaa......................................................................................................................................................................................................................   1   0   JCK-3
    ..............uagaggacaaauucaauuugaU......................................................................................................................................................................................................................   2   1   LT-1
    ..............uagaggacaaauucaauuugaa......................................................................................................................................................................................................................   1   0   JCK-1
    ...............agaggacaaauucaauuuga.......................................................................................................................................................................................................................   1   0   LT-3
    ...............agaggacaaauucaauuugaac.....................................................................................................................................................................................................................   2   0   LT-1
    ................gaggacaaauucaauuugaac.....................................................................................................................................................................................................................   2   0   JCK-1
    ................gaggacaaauucaauuugaacA....................................................................................................................................................................................................................   1   1   LT-3
    ....................acaaauucaauuugaacucuacc...............................................................................................................................................................................................................   1   0   LT-1
    ....................acaaauucaauuugaacucuacca..............................................................................................................................................................................................................   1   0   JCK-2
    ....................acaaauucaauuugaacucuacca..............................................................................................................................................................................................................   1   0   JCK-3
    .....................caaauucaauuugaacucuacc...............................................................................................................................................................................................................   1   0   JCK-1
    .....................caaauucaauuugaacucuacc...............................................................................................................................................................................................................   1   0   LT-2
    .....................caaauucaauuugaacucuacc...............................................................................................................................................................................................................   1   0   LT-1
    .....................caaauucaauuugaacucuacc...............................................................................................................................................................................................................   1   0   LT-3
```

Mature Star

```
agaaucuaucaugauagaggacaaauucaauuugaacucuaccaccuuaggagaagucaaguucaguuugaacucuacuuuagaagaauucaauuucaaguugaacucuaccaaaggcgaauucaauuucaauuugaauuccaccaccucuucuccuuuauauauaugcauuuaggcuuauuguaaaauuugaucccauuuuguaaaaagagagagcuuuaauucucuuccucucaauuauauugucuug
.....................caaauucaauuugaacucuacU...............................................................................................................................................................................   1   1   LT-1
.....................caaauucaauuugaacucuacca..............................................................................................................................................................................   2   0   JT-3
.....................caaauucaauuugaacucuacca..............................................................................................................................................................................   27  0   LCK-1
.....................caaauucaauuugaacucuacca..............................................................................................................................................................................   3   0   LT-2
.....................cCaauucaauuugaacucuacca..............................................................................................................................................................................   1   1   LCK-1
.....................caaauucaauuugaacucuacca..............................................................................................................................................................................   7   0   JT-2
.....................caaauucaauuugaacucuacca..............................................................................................................................................................................   1   0   JCK-2
.....................caaauucaauuugaacucuacca..............................................................................................................................................................................   1   0   LT-1
.....................caaauucaauuugaacucuacca..............................................................................................................................................................................   25  0   LCK-2
.....................caaauucaauuugaacucuacca..............................................................................................................................................................................   1   0   JCK-3
.....................caaauucaauuugaacucuaccac.............................................................................................................................................................................   1   0   JCK-3
.....................caaauucaauuugaacucuaccacA............................................................................................................................................................................   2   1   JCK-2
.....................caaauucaauuugaacucuaccacc............................................................................................................................................................................   1   0   JCK-1
.....................caaauucaauuugaacucuaccacc............................................................................................................................................................................   2   0   JCK-3
.....................caaauucaauuugaacucuaccacA............................................................................................................................................................................   1   1   JCK-1
......................aaauucaauuugaacucuacc...............................................................................................................................................................................   1   0   JCK-1
......................aaauucaauuugaacucuacca..............................................................................................................................................................................   5   0   JCK-2
......................aaauucaauuugaacucuacca..............................................................................................................................................................................   3   0   JCK-3
......................aaauucaauuugaacucuacca..............................................................................................................................................................................   2   0   LT-3
......................aaauucaauuugaacucuacca..............................................................................................................................................................................   2   0   JCK-1
......................aaauucaauuuNaacucuaccacc............................................................................................................................................................................   1   1   JCK-1
......................aaauucaauuugaacucuaccacc............................................................................................................................................................................   3   0   JT-3
......................aaauucaauuugaacucuaccacc............................................................................................................................................................................   67  0   JCK-3
......................aaauucaauuugaacucuaccacc............................................................................................................................................................................   2   0   JT-1
......................aaauucaauuugaacucuaccacc............................................................................................................................................................................   6   0   LT-1
......................aaCuucaauuugaacucuaccacc............................................................................................................................................................................   1   1   JCK-1
......................aaauucaauuugaCcucuaccacc............................................................................................................................................................................   1   1   JCK-3
......................aaCuucaauuugaacucuaccacc............................................................................................................................................................................   1   1   JCK-2
......................aaauucaauuugaacucuaccacc............................................................................................................................................................................   59  0   JCK-1
......................aaauucaauuugaacucuaccacc............................................................................................................................................................................   90  0   JCK-2
......................aaauucaauuugaacucuaccacc............................................................................................................................................................................   10  0   LT-3
......................aaauucaauuugaacucuaccacc............................................................................................................................................................................   3   0   JT-2
......................aCauucaauuugaacucuaccacc............................................................................................................................................................................   1   1   JCK-3
......................aaauucaauuugaacucuaccaccA...........................................................................................................................................................................   4   1   LT-1
.......................aauucaauuugaacucuacca..............................................................................................................................................................................   1   0   JCK-3
.......................aauucaauuugaacucuacca..............................................................................................................................................................................   1   0   JCK-1
.......................aauucaauuugaacucuacca..............................................................................................................................................................................   2   0   LT-1
.......................aauucaauuugaacucuaccac.............................................................................................................................................................................   1   0   JCK-2
.......................aauucaauuugaacucuaccacc............................................................................................................................................................................   1   0   JCK-2
.......................aauucaauuugaacucuaccacc............................................................................................................................................................................   1   0   JCK-3
.......................aauucaauuugaacucuaccaccu...........................................................................................................................................................................   20  0   JCK-2
.......................aNuucaauuugaacucuaccaccu...........................................................................................................................................................................   1   1   JCK-3
.......................aauucaauuugaacucuaccaccu...........................................................................................................................................................................   12  0   JT-1
.......................aauucaauuugaacucuaccaccu...........................................................................................................................................................................   13  0   JCK-3
.......................aauucaauuugaacucuaccaccu...........................................................................................................................................................................   11  0   JCK-1
.......................aauucaauuugaacucuaccaccu...........................................................................................................................................................................   1   0   LT-1
........................auucaauuugaacucuacc...............................................................................................................................................................................   4   0   LT-2
........................auucaauuugaacucuaccU..............................................................................................................................................................................   27  1   LCK-1
........................auucaauuugaacucuaccU..............................................................................................................................................................................   12  1   LCK-2
........................auucaauuugaacucuaccac.............................................................................................................................................................................   1   0   JCK-1
........................auucaauuugaacucuaccac.............................................................................................................................................................................   2   0   JCK-3
........................auucaauuugaacucuaccac.............................................................................................................................................................................   2   0   JCK-2
........................auucaauuugaacucuaccacc............................................................................................................................................................................   6   0   JCK-2
........................auucaauuugaacucuaccacA............................................................................................................................................................................   1   1   LT-3
........................auucaauuugaacucuaccacc............................................................................................................................................................................   11  0   JCK-3
........................auucaauuugaacucuaccacc............................................................................................................................................................................   8   0   JCK-1
........................auucaauuugaacucuaUcacc............................................................................................................................................................................   1   1   JCK-2
........................auucaauuugaacucuaccacA............................................................................................................................................................................   1   1   JCK-1
........................auucaauuugaacucuaccacA............................................................................................................................................................................   2   1   LT-2
........................auucaauuugaacucuaUcacc............................................................................................................................................................................   1   1   JCK-3
........................auucaauuugaacucuaccaccu...........................................................................................................................................................................   2   0   JCK-2
........................auucaauuugaacucuaccaccu...........................................................................................................................................................................   2   0   LT-3
........................auucaauuugaacucuaccaccu...........................................................................................................................................................................   5   0   JCK-3
........................auucaauuugaacucuaccaccu...........................................................................................................................................................................   2   0   LT-1
........................auucaauuugaacucuaccaccu...........................................................................................................................................................................   6   0   LCK-3
........................auucaauuugaacucuaccaccuu..........................................................................................................................................................................   24  0   JCK-2
........................auucaauuugaacucuaccaccuu..........................................................................................................................................................................   13  0   JCK-3
........................auucaauuugaacucuaccaccuu..........................................................................................................................................................................   18  0   JCK-1
.........................uucaauuugaacucuacca..............................................................................................................................................................................   3   0   LT-2
.........................uucaauuugaacucuaccac.............................................................................................................................................................................   2   0   JCK-1
```

```
                     Mature                   Star
agaaucuaucaugauagaggacaaauucaauuugaacucuaccaccuuaggagaagucaaguucaguuugaacucuacuuuagaagaauucaauuucaaguugaacucuaccaaaggcgaauucaauuucaauuugaauuccaccaccucuucuccuuuauauauaugcauuuaggcuuauuguaaaauuugaucccauuuuguaaaaagagagagcuuuaauucucuuccucucaauuauauugucuug
.........................uucaauuugaacucuaccacc............................................................................................................................................................................................................    1    0    JCK-2
.........................uucaauuugaacucuaccacc............................................................................................................................................................................................................    2    0    LT-1
.........................uucaauuugaacucuaccacc............................................................................................................................................................................................................    2    0    JCK-3
.........................uucaauuugaacucuaccacc............................................................................................................................................................................................................    2    0    LT-3
.........................uucaauuugaacucuaccaccu...........................................................................................................................................................................................................    8    0    JCK-3
.........................uucaauuugaacucuaccaccA...........................................................................................................................................................................................................    1    1    JCK-3
.........................uucaauuugaacucuaccaccu...........................................................................................................................................................................................................    8    0    JCK-1
.........................uucaauuugaacucuaccaccu...........................................................................................................................................................................................................    2    0    LT-2
.........................uucaauuugaacucuaccaccu...........................................................................................................................................................................................................    12   0    JCK-2
.........................uucaauuugaCcucuaccaccu...........................................................................................................................................................................................................    1    1    JCK-3
..........................ucaauuugaacucuacca..............................................................................................................................................................................................................    1    0    LT-3
..........................ucaauuugaacucuaccaccuu..........................................................................................................................................................................................................    1    0    JCK-3
..........................ucaauuugaacucuaccaccuu..........................................................................................................................................................................................................    1    0    JCK-2
...........................caauuugaacucuaccaccG...........................................................................................................................................................................................................    1    1    JCK-2
...........................caauuugaacucuaccaccu...........................................................................................................................................................................................................    5    0    JCK-2
...........................caauuugaacucuaccaccu...........................................................................................................................................................................................................    9    0    JT-3
...........................caauuugaacucuaccaccu...........................................................................................................................................................................................................    3    0    JCK-1
...........................caauuugaacucuaccaccu...........................................................................................................................................................................................................    3    0    JT-2
...........................caauuugaacucuaccaccu...........................................................................................................................................................................................................    8    0    JCK-3
...........................caauuugaacucuaccaccu...........................................................................................................................................................................................................    5    0    LCK-3
...........................caauuugaacucuaccaccuu..........................................................................................................................................................................................................    5    0    JCK-3
...........................caauuugaacucuaccaccuu..........................................................................................................................................................................................................    3    0    JCK-2
...........................caauuugaacucuaccaccuu..........................................................................................................................................................................................................    1    0    LT-1
...........................caauuugaacucuaccaccuu..........................................................................................................................................................................................................    2    0    JCK-1
............................aauuugaacucuaccaccu...........................................................................................................................................................................................................    1    0    LT-1
............................aauuugaacucuaccaccuu..........................................................................................................................................................................................................    1    0    JCK-3
............................aauuugaacucuaccaccuu..........................................................................................................................................................................................................    1    0    LT-3
.......................................................................................................................................gaauuccaccaccucuAcuc...............................................................................................    1    1    JCK-2
..................................................................................................................................................ccucuucuccuuuauauauaug....................................................................................    1    0    JCK-3
........................................................................................................................................................................................................................cuuuaauucucuuccucucaauuGuauug.....    1    1    JCK-2
........................................................................................................................................................................................................................cuuuaauucucuuccucucaauuGuauug.....    2    1    JCK-3
............................................................................................................................................................................................................................aauucucuuccucucaauuGuauugucuug    2    1    JCK-3
............................................................................................................................................................................................................................aauucucuuccucucaauuGuauugucuug    2    1    JCK-2
```
